# Risk Factors and Current Health-Seeking Patterns of Migrants in Northeastern Mexico: Healthcare Needs for a Socially Vulnerable Population

**DOI:** 10.3389/fpubh.2015.00191

**Published:** 2015-08-06

**Authors:** Philippe Stoesslé, Francisco González-Salazar, Jesús Santos-Guzmán, Nydia Sánchez-González

**Affiliations:** ^1^Department of Social Sciences, University of Monterrey, Monterrey, Mexico; ^2^Mexican Social Security Institute (IMSS), Monterrey, Mexico; ^3^Department of Basic Sciences, University of Monterrey, Monterrey, Mexico; ^4^School of Medicine, Monterrey Institute of Technology and Higher Education, Monterrey, Mexico; ^5^Faculty of Nutrition and Public Health, Autonomous University of Nuevo León, Monterrey, Mexico

**Keywords:** risk factors, undocumented migrants, social vulnerability, tuberculosis knowledge and perceptions, barriers to health

## Abstract

This study identified risk factors for health and access to healthcare services of migrants during their journey across Mexico to the United States. Data were collected in shelters located in Monterrey, the largest city of northeastern Mexico, through a basic clinical examination and a survey completed by 75 migrants; 92% of them were undocumented Central Americans. During their transit, they are at a high risk of contracting, developing, and transmitting diseases. The need of working to survive affects health-seeking behavior and a constant fear of being traced keeps migrants away from public health services, which delays diagnosis and treatment of diseases. Negligent lifestyles, such as smoking, drinking (31.8% of men and 11.1% of women), and drug abuse (13% of men and 11% of women), were found. Regarding tuberculosis (TB), undocumented migrants are usually not screened, even though they come from countries with a high TB burden. Besides, they might be overexposed to TB because of their living conditions in overcrowded places with deficient hygiene, protection, and malnutrition (54.7% of the sample). Possible comorbidities like acquired immune deficiency syndrome (AIDS; 4%) and diabetes (2.7%, but probably under-diagnosed) were referred. Migrants have little TB knowledge, which is independent of their level of education or a previous experience of deportation. About one-third of the migrants were totally unfamiliar with TB-related symptoms, while 36% had correct knowledge of basic TB symptoms. We conclude that a shortage of information on the highly vulnerable migratory population combined with a lack of social support and health education among migrants may play a significant role in the spread of communicable diseases. We recommend that health authorities address this urgent, binational, public health concern in order to prevent outbreaks of emerging infections.

## Introduction

In 2013, 214 million migrants lived for at least 1 year in a country different from their home country (representing 3.1% of the world population), against 150 million migrants in 2000 (1). Apart from a rapidly increasing migration number, migration also involves a wider diversity of cultural groups. About 82 million people per year migrate from a developing country to a developed one. Furthermore, the latest estimations suggest that around one-third of migration from developing countries could be irregular (2). With migration being a growing tendency, migrants experience a unique diversity in health needs and profiles.

Worldwide, migrants are more vulnerable than nationals, as they have fewer rights than people with a citizenship. In Mexico, the life style, journey, and work of migrants, especially the undocumented migrants from Central and South America, lead to considerable risks to personal, physical, and mental well-being (3–5). Migrants’ well-being is further compromised by irregular access to social and health services, immigrant status, and a prevailing anti-migrant attitude of the general public (6–8). Indeed, legal and practical obstacles limit access to health services on both sides of the Mexican–United States (US) border, and the consequential late detection of illnesses negatively affects the health of immigrants (9).

Transnational migration through Mexico affects the public health situation by connecting areas with diverse disease prevalence and other socioeconomic factors. Most of these health issues represent challenges for the limited territorial public health systems, because migrants are by definition mobile and hard-to-reach persons. Thus, the interest of our research is to determine to what extent the migration process affects the health of both the migrants and the general population. Hereto, the present pilot study aims to identify the risk factors of illnesses among migrants, as well as to get insight into the health-seeking patterns and TB knowledge among North, Central, and South American (im)migrants who pass through the city of Monterrey, Mexico, in their quest to reach the US. To our knowledge, this is the first report on current patterns of migration and their impact on health in this specific population in northeastern Mexico. Our results provide a basis for rethinking the specific healthcare needs in order to protect the health status of the general population.

## Materials and Methods

This study is an observational, transversal, and descriptive study realized from September 1st to November 30th, 2014, at temporary shelters for migrants in Monterrey. Data were collected through a basic clinical examination and a survey.

### Population definition

We adopted the definition of the International Organization for Migration’s definition for “undocumented immigrants”: “Persons who change their place of usual residence” but without having a legal residence situation (10).

### Ethics and consent

The research protocol followed principles of the Declaration of Helsinki and was approved by the University of Monterrey under the project code UIN15012. Written informed consent was obtained from all participants. In case of minors, informed consent was obtained from the parents or accompanying adults. No unaccompanied minor participated in the study.

### Sample

The sample consisted of 75 temporal immigrant volunteers of any age and gender who attended one of the two migrant shelters managed by the Catholic Church in the Monterrey Metropolitan Area; the “Centro de Apostolado San Nicolás de Tolentino – CasaNicolás” and the “Casa Santa Martha.” It is important to underline that in México the shelters for undocumented migrants are mostly managed by different churches, the majority of them were managed by the Catholic Church. Rather than realizing evangelization, these shelters provide help and assistance to the (im)migrants. They do this independent of their religious beliefs or those of the migrants. Therefore, we consider that the formal religious characteristic of the shelters does not affect the origin or representativeness of the population sample.

Subjects with incomplete information or immigrants who dropped out the study before completion were not considered. The sample encompassed 71 undocumented South and Central American immigrants and 4 North Americans. We decided to include the latter because they complied with the definition of migrant and suffered the same mental and physical health stressors and social vulnerability similar to undocumented immigrants (11) (Table 1).

**Table 1:** **Demographic data**.

Variable	Observation	Frequency
Gender (males)	75	66 (88%)
Age in years (mean ± SD)	75	32.5 ± 1.1
**Migrant shelter**		
CasaNicolás	64	85.3%
Casa Santa Martha	13	14.7%
**Religion**		
Catholic	28	37.3%
Protestant	27	36.0%
Other	2	2.7%
None	13	17.3%
No answer	5	6.7%
**Nationality**		
Honduras	41	54.6%
Guatemala	15	20.0%
El Salvador	12	16.0%
Mexico	3	4.0%
Ecuador	1	1.3%
USA	1	1.3%
Nicaragua	1	1.3%
Venezuela	1	1.3%
**Marital status**		
Single	40	53.3%
Cohabiting	20	26.7%
Married	12	16.0%
Divorced	3	4.0%
**Education level**		
Elementary		
Incomplete	15	20.0%
Complete	25	33.3%
High school		
Incomplete	3	4.0%
Complete	17	22.7%
University or technical carrier	9	11.9%
None	6	8.0%

### Sampling location

Monterrey is a cosmopolitan city located in northeastern Mexico and is the capital city of the state of Nuevo Leon. Over 4.4 million people live in this metropolitan area. It is one of the most important cities of the country and it represents one of the traditional routes of migrants coming from southern states of Mexico and from the other Central and South American countries (12).

### The questionnaire

Our team, which included experts in TB and migration, designed a semi-structured, Spanish questionnaire with closed and open-ended questions, which included quantitative and qualitative methods. The questionnaire was based on previous national and international validated questionnaires (13–18), recommendations from a literature review (19), and the guidelines of the WHO “Tuberculosis PREVALENCE SURVEYS: handbook” (20).

To ensure that the survey is in accordance with the education level of the participants, the questionnaire was adapted to the education level specified in the Program of the last level of the Elementary School in Mexico (21). The questions were focused on specific topics, contained congruency control, and were limited in scope to avoid exhaustion of the participants; all to promote the reliability of the responses. A draft version was piloted in a 12-person group, and pointless, duplicated, or unsuitable questions were eliminated. The resulting succinct, clear, and unequivocal questionnaire was reorganized into logical sections and standardized. The reliability of the survey was confirmed by supplying the survey questionnaire twice to the same individuals with a long enough time difference to make it highly unlikely the respondents remember their first responses. Translation from Spanish to English was used once.

### Procedures

The survey was applied at the shelters during 45-to-60-min, face-to-face interviews in a relaxed atmosphere, which enabled to clarify the eventual unclear answers. Random surveys were cross-checked for continuous reliability. As a result, we collected a limited, but consistent and trustworthy amount of information, which was analyzed for the following characteristics:

1. Socio-demographic profile (age, sex, ethnicity, place of origin, education, and occupation).
2. Reasons for immigration and history of their journey (transports used, type of housing, and experiences with authorities and criminal groups).
3. Medical history.
4. Personal habits and lifestyle (tobacco, alcohol, and substance use).
5. Baseline dietary assessment.
6. Emotional well-being and social support.
7. Perceived barriers to health services and health-seeking practices.
8. Basic TB knowledge and attitudes.

In the same session, we determined anthropometric measures (height, weight, waist, and hip circumference) and blood pressure. Furthermore, we performed walk-through inspections of the shelters, to be familiar with the dormitories and the living spaces. Data collection and interpretation followed confidentiality procedures.

### Data analysis

All data were collected in an Excel Spreadsheet and subsequently fed into a Stata Software v11.0 database (College Station, TX, USA). The data were then coded and displayed according to Miles and Huberman’s methods (22), before being processed through descriptive statistical analyses. Outcomes were reported as means ± standard deviation (SD) or frequencies.

## Results

In our sample, most of the immigrants were males (88%) and their mean age was 32 ± 11.1 years (range: 14–60 years); the predominant age group was from 20 to 30 years (43%). More than half of the migrants came from Honduras (54.6%), whereas other Central American countries (Guatemala, El Salvador, and Nicaragua) represented 37.3% of the sample (Table 1).

As a matter of fact, this sample is consistently similar to the total population of undocumented immigrants in the Monterrey Metropolitan Area during the year 2014, according to the annual report of CasaNicolás (the unique liable and available source of information on undocumented migrants). In fact, during 2014, CasaNicolás studied 87% of male immigrants including 63% from Honduras (23).

More than half of the volunteers (53%) had only elementary school education, either complete or incomplete. One-third (25 subjects, 34%) had high school or baccalaureate education, and only four subjects (5.3%) had higher level education, of either a technical or professional career. Religion and marital status are reported in Table 1.

The weight, height, and body mass index (BMI) of the participants were 69.6 ± 10.6 kg, 164.7 ± 8.5 cm, and 25.7 ± 3.9 (mean ± SD), respectively. Almost half of the subjects were overweight (46.6%) or obese (10.6%). None of them was underweight, despite the fact that more than half of the immigrants referred diminished or limited food intake during their journey (Table 2).

**Table 2:** **Nutritional assessments and personal habits**.

Variable	Observation	Mean **±** SD
**Weight (kg)**		
Men	66	67.8 ± 9.1
Women	9	72.8 ± 15.9
**Height (cm)**		
Men	66	166 ± 7.3
Women	9	155 ± 9.1
Body mass index	45	25.7 ± 4.0
Underweighted (<18.5)	0	0
Normal weight (18.5–24.9)	35	46.6%
Overweighed (25–29.9)	35	46.6%
Obesity (>30)	5	10.6%
**Waist (cm)**		
Men	63	87.6 ± 9.5
Women	9	100.7 ± 11.9
**Hips (cm)**		
Men	27	98.6 ± 6.5
Women	6	107 ± 12.4
**Hygiene (interviewer criteria)**		
Good	56	74.7%
Regular	10	13.3%
Bad	7	9.3%
No answer	2	2.7%
**Alcohol**		
Men	21	31.8%
Women	1	11.1%
**Tobacco**		
Men	21	31.8%
Women	1	11.1%
Drugs		
Men	9	12.0%
Women	1	1.3%
**Food intake during last month**		
Increased	12	16.0%
Equal	22	29.3%
Diminished	35	46.7%
Limited	6	8.0%

With respect to personal habits, the subjects presented good hygiene (74.7%), but alcohol and tobacco consumptions were referred in the same proportions: 31.8% and 11.1% of males and females, respectively. Almost half of them (44.8%) had started drinking and 80% had started smoking as teenagers. Among males, 38% consumed both alcohol and tobacco, whereas only 11.1% of the females consumed both. Illegal drugs were referred in 13.6% of males and 11% of females. Of them, 66% referred using marihuana at least once and 22% referred cocaine (Table 2).

Moreover, 10.7% of men and 22.8% of women mentioned having health problems, with upper respiratory disease being the most common (15.2%); there were trauma or lesions in 6.1%, gastrointestinal disease in 4.6%, and urinary disease in 3% of the sample. More women than men had medical insurance (22.2% and 12.1%, respectively). Most preferred public health services (22.7%) and 14.7% did not seek medical attention even when sick (Table 3).

**Table 3:** **Health status**.

Variable	Observation	Frequency (%)
**Health issues**		
Men	63	10.7
Women	8	22.8
**Diseases**		
Respiratory (upper)	10	15.2
Gastrointestinal	3	4.6
Urinary	2	3.0
Trauma & Lesions	4	6.1
Other	3	4.6
No answer	53	66.7
Medical insurance	10	13.3
% of men with insurance	8	12.1
% of women with insurance	2	22.2
**Frequency of contact with healthcare system**		
≥2 times over the past year	19	25.3
Once over the past year	12	16.0
1–2 over the past 5 years	8	10.7
Once over the past 5 years	11	14.7
Never in the past 5 years	24	32.0
No data	1	1.3
**Type of health service sought when necessary (various possible answers)**		
Public med. in home country	8	10.7
Public medicine in Mexico	7	9.3
Public medicine in the US	2	2.7
Private medicine in Mexico	1	1.3
Migrant shelter	3	4.0
Drugstore	8	11.0
Self-medicated	34	45.0
No medical attention	11	14.7
No answer	42	56.0
**High blood pressure**		
Men		17.0
Women		20.0
**Tuberculosis**		
BCG vaccine	74	98.7
TB	75	1.3
TB contacts	75	14.7
**Known TB contact**		
Yes	11	14.7
No	64	85.3
**AIDS**	3	4.0
**Diabetes**	2	2.7

Twenty percent of women and 17% of men had high blood pressure, and most of them declared to have had visited the healthcare system rarely. One quarter (25.3%) affirmed having been in touch with the healthcare system two or more times over the past year, and 12 of them (16%) once, but more than half (57.4%) had contacted less than once over the past year. In addition to this poor pattern of healthcare seeking behavior, 14.7% referred to be close to a patient with TB, 4% referred to have acquired immune deficiency syndrome (AIDS) and 2.7% diabetes mellitus (DM); 98.7% referred having received the Bacillus Calmette–Guérin (BCG) vaccine (Table 3).

Participants understood that TB is a disease associated with significant morbidity and mortality, and 81.3% of the subjects considered TB a serious or very serious disease. While 17.3% mentioned vague symptoms that may relate to any kind of discomfort; only 30.7% correctly identified coughing for more than two weeks and 13.3% mentioned fever for more than one week, but none mentioned shortness of breath and weight loss. Also, 30.7% mentioned at least one wrong symptom and 33.3% had no idea at all (Table 4). Besides, less than one-fifth (18.7%) thought they were infected. Physicians and television were cited as the two most important sources of information on TB (Table 4).

**Table 4:** **TB knowledge and perception**.

Variable	Observation	Frequency (%)
**Can you contract TB**	75	18.7
**TB symptoms knowledge**		
Correct answer	27	36.0
Incorrect answer	23	30.7
Does not know	25	33.3
**TB symptoms knowledge without misconceptions (various possible answers)**		
Cough >2 weeks	23	30.7
Fever >1 week	10	13.3
Vague symptoms	13	17.3
Shortness of breath	0	0.0
Weight loss	0	0.0
**How serious is TB**		
Very serious	45	60.0
Serious	16	21.3
Not too serious	1	1.3
Do not know	13	17.3

It was the first migratory journey for 57.5% of our sample. Poverty and economic reasons had been the motive of emigration for the majority (65.3%), violence for 22.7%, family reunion for 8%, and political persecution for 4%. A quarter had had a previous undocumented experience and 21.3% had been deported from the US back to their home country (Table 5). For over half of them (53%), the journey’s endpoint was the US; the majority of them (90.7%) had an exact destination.

**Table 5:** **Emigration Experiences**.

Variable	Observation	Frequency (%)
**Reason for emigration (various possible answers)**		
Poverty and economic reasons	75	65.33
Violence	75	22.66
Family reunion	75	8.00
Political persecution	75	4.00
Other reasons	75	13.33
**First immigration travel**	73	57.53
**Previous experience in the US as an undocumented migrant**	19	25.33
**Previously deported from Mexico to home country**	2	2.66
**Previously deported from the US to home country**	16	21.33
**Final destination**	73	57.53
USA	40	53.30
Other	9	12.00
No answer	26	34.60
**Place to go in the US**	68	90.70
**Problems during travel (various possible answers)**		
Criminal organization	17	22.60
Police/migration	28	37.30
“Pollero” scam	4	5.30
Others (civil population)	4	5.30
None	18	24.00
No answer	9	12.00
**Type of problem (various possible answers)**		
Violent assault	20	26.66
Oral and physical threats	15	20.00
Extortion	20	26.66
Kidnapping	6	8.00
Other kind of problems	11	14.66
**Journey funding (various possible answers)**		
Savings	46	61.33
Temporary work	23	30.66
Assistance (NGO/local population)	15	20.00
Help of the shelters	24	32.00
Other	12	16.00
**Had to borrow money?**		
Yes	21	28.00
No	45	60.00
No answer	9	12.00

Almost two-thirds (64%) reported problems during their travel, most often with the authorities (37.3%), criminal organizations (22.7%), and with the smugglers (“polleros”; 5.3%). Over a quarter (26.7%) reported violent assaults and a similar percentage had suffered economic extortions. Verbal and physical threats were reported by 20% of the cases and 8% of the migrants mentioned being victims of kidnapping.

To fund their journey, 28% had borrowed money from a relative or friend and 61.3% had used their savings, but most depended on work or financial support during their trip (Table 5).

## Discussion

### Socio-demographic general overview

Undocumented migration represents 5–30% of the total immigrant population depending on the country (24). Unauthorized migration through Mexico is a major and growing issue for both Mexico and the US. The number of undocumented migrants in the US increased from around 3 or 4 million in 1993 to 12.2 million in 2007, and 11.2 million in 2012 (25). In US, the Latin American undocumented immigrants have greater problems to access the healthcare system and consequently use it less than undocumented immigrants from other parts of the world (26). However, the exact burden of undocumented immigrants in northeastern Mexico and the impact on public health are not known.

The socio-demographic profile of our population sample is in line with the available data on the 1,112 persons who attended the CasaNicolás between February and October 2014; the others’ shelters have not been registered. Most immigrants were male (88% in our sample and 73% in the CasaNicolás register) and from Honduras (55% and 63%, respectively). Together with other Central American countries, they represented 92% of our sample (Table 1), versus 84% of the shelter’s total population.

Likewise, the reasons for migration are similar between our 75-person sample and the total population of CasaNicolás. In both the groups, poverty and economic reasons were the most frequent causes of emigration: unemployment, salaries too low to mitigate high inflation, debts, and economic crisis (65.33% of our sample and 61% of the total CasaNicolás population). Violence was mentioned in second place (22.66% of our sample; 7% of the total population), followed by family reunion (8 and 2%, respectively) (Table 5).

### Migration and health interaction

Monterrey is an economically growing city where the undocumented immigrants live temporarily and where they usually do not struggle to find a job. However, this population lacks adequate healthcare due to their immigrant status. Accordingly, their only access to healthcare generally limited to some local clinics at best, when they accept to treat them for free.

Migration and health interact and cause a decline in the general health of migrants. Undoubtedly, migrants' health depends on individual characteristics (gender, age, education, substance use, etc.), but it is also determined by contextual factors that cover far more than the individual health aspects. The migrant’s well-being is vulnerable because of a complex interaction among the aforementioned individual characteristics, lifestyles (e.g., dehydration, habits, and food shortages), personal beliefs and attitudes, living and working conditions, and environmental characteristics (27).

Migrants face complex problems ranging from housing shortage to overcrowded shelters with poor ventilation, propitious conditions for the transmission of diseases (TB, influenza, fungi). They are also confronted with cultural and psychosocial obstacles, such as poor education and knowledge on health issues, which impede their access to health services. Another challenge is maintaining their personal safety and security within an unfriendly environment that combines food insecurity, social vulnerability in general, and overexposure to violence. When arrested by the Mexican or US migration authorities, they are usually imprisoned in overcrowded places that serve as incubators for contagious diseases and infections (28). Lastly, some of their behaviors, like untreated substance abuse and mental illness, might affect their physical health status.

Smoking, and alcohol and substance abuse are known risk factors for many diseases, including infections, because they compromise the immune response. However, adopting healthier habits may be harder for migrants than for the general population, because they have to handle much more stressful situations they rely with the use of tobacco, alcohol, and substances (29). For this reason, migrants need special counseling and medication to help them quit.

Regarding TB, we suspected TB in a volunteering migrant, but chest radiography excluded such a diagnosis. Still, TB is an excellent illustration of the complexity of healthcare issues in relation to migration. The failure to detect TB, or its late diagnosis, and the inadequate treatment follow-up in migratory TB patients precisely reflect the underlying educational/cultural factors (absence of information), economical (shortage of resources for transportation and housing), and psychosocial factors (e.g., feeling ashamed or guilty about the disease, or failure to establish a strong doctor–patient relationship), and several other social barriers (professional, familiar, etc.) (30–32).

TB prevalence is higher in socially vulnerable populations (33–35). Our study displays the high burden of health issues in the undocumented immigrant population, with pronounced conditions of social vulnerability and epidemiological risks. Undocumented migrants should be considered a focal point of health attention for being overexposed to risk factors, especially in the case of AIDS patients (4% prevalence in our population against 0.2% in the general population of Nuevo Leon in 2013) (36) and migrants suffering diabetes (only 2.7% of our sample).

### The living conditions during transit as a complication to health access

Since migrants usually face structural socioeconomic needs (which is the most common reason for migration), they tend not to spend money on their health. In most cases, cough, weight loss, and tiredness are considered normal and not a reason to seek medical care; 32% of our sample had no contact with any healthcare system in the past 5 years and more than half (57.4%) had not seen a physician at least once over the past year (Table 3). Regular medical consults were uncommon under normal conditions, but are even more unlikely during their migration process. This unfortunate habit, combined with a poor knowledge on health issues, increases the delay in health seeking.

Moreover, undocumented Central American immigrants in northeastern Mexico are undoubtedly a hard-to-reach population. By definition, they are a “moving population”. Their itinerant lifestyle complicates the detection of diseases and the eventual follow-up of any treatment. Their relatively bad health condition (almost one-third with a health issue of any kind and 78% reported at least one dental problem in the past 6 months) can be explained by: (1) the accumulation of risk factors during their trip (e.g., overcrowding, malnutrition, violence; women being particularly vulnerable), (2) fear of deportation, and (3) the uncertainty about where to access healthcare.

As compared to legal immigrants, undocumented migrants are more vulnerable, face more inequality situations based on the economic, political, and institutional frameworks, and confront higher structural barriers (37). Understanding the situation of an undocumented immigrant journey enables the understanding of health inequalities. Many migrants had had limited access to the health system in their home countries due to their socioeconomic limitations on the one hand and the scarcity of community healthcare facilities on the other hand. Also during their stay in Mexico, they remain outside mainstream social systems (e.g., housing, work, health) and may suffer major social stigma and psychological issues. The same might be said for TB. Migrants not only have a greater risk of prior infection (38), but are also exposed to a high risk during the transit. For this reason, the transnational strategies developed to address the situation of TB in the border should be applied during every step of the migration process (origin, transit, temporary stays, final destination, and eventual return) and overcome territorial boundaries.

Finally, insufficient and inadequate nutrition is an important issue affecting health during the migration process. Due to insufficient economic strength and lack of physical liberty, the food consumed by migrants does not cover their dietary needs or hygiene and safety aspects (Table 2). Only during stays at temporary shelters like CasaNicolás or Casa Santa Martha, they get balanced and quantitatively sufficient food that provides adequate intake of protein, fat, and carbohydrates. A healthy diet is important to improve and guarantee an immigrant’s general condition.

### Living with the fear of deportation

In 2014, the *Instituto Nacional de Migración* (INM) registered 127,149 immigrants that had been presented to the Mexican migratory authorities, of which 1,477 in Nuevo Leon. Most of them were Central Americas: 119,714 (94%) and 1,365 (92%), respectively (39). The main concern for undocumented migrants is their legal status, as they are subject to deportation when they are caught. They are not only exposed to violence in the streets with physical and mental consequences, but they also feel the stigma of being perceived as possibly dangerous people, addicts, or burglars. Furthermore, they are far more vulnerable to be exploited, socially marginalized, and discriminated in everyday life, including their access to health services. It is well-known that fear of deportation withholds them from seeking health support (40–42). They usually associate the healthcare facilities with a place that exposes their undocumented status. The control of infectious disease and the decrease of community risk will only be possible by including the undocumented immigrants into the public health programs without discriminating them because of their legal status.

### An overexposure to violence

Generally speaking, since the beginning of the “Mexican Drug War” in 2006, which empowered the criminal groups, vulnerable immigrants are one of the main victim groups, especially in the northeastern states of Tamaulipas, Coahuila, and Nuevo Leon. Organized crime has increased personal insecurity among both the undocumented migrants and Mexican emigrants.

In addition, the consciousness of their own social stigmatization discourages migrants to look for help when required. Their illegal status interferes with medical follow-up, as it implies providing personal and delicate information that may identify them as subjects for deportation. Moreover, the psychological trauma of violent episodes lived during their migration process (64% reported some kinds of problems – i.e., extortions, assaults, and kidnapping) might provoke mistrust on the use of their personal information.

Considering, furthermore, that they are separated from their families and are without social networks to provide protection and help when needed, they are more likely to fall into alcohol and substance abuse. For all the above, we recommend offering them systematic, psychological support in the shelters. Psychological support should be focused on solving depressive disorders and socio-emotional problems and on establishing commitment to personal healthcare issues. This kind of attention would help the immigrants to understand their health issues and to consequently develop the search of medical attention when necessary and to improve the adherence to eventual treatments when sick (especially, in lengthy treatments such as the one required for TB).

### Socioeconomic barriers

Despite the fact that most of them are below the line of poverty, migrants have to develop financial strategies to cover journey expenses (mainly transport and food), even if this implies deepening the financial burden they structurally suffer. In our sample, more than half depended to a certain extent on others: 20% benefited from the assistance of the general population or social organizations that support migrants, and 32% from the punctual help of the shelters that gave them some money to cope with the economic cost of their journey. Besides, almost one-third (28%) had arranged a loan before their departure to cover (Table 5). This economic dependence increases their vulnerability.

**Figure 1:**
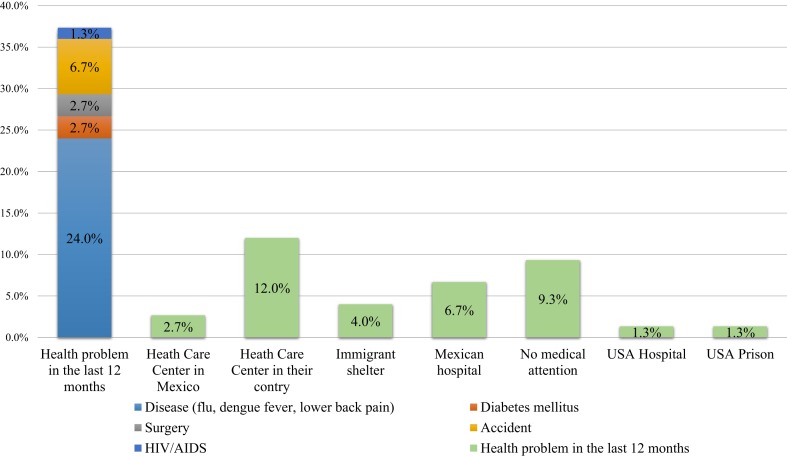
**Type of health problems in the past 12 months and medical attention received**.

Poverty and inequality are interconnected, and the most vulnerable groups represent a huge health challenge (43). Apart from their illegal status, socioeconomic barriers were the main obstacles for proper healthcare seeking in our sample. In this respect, our data confirm similar results reported for (undocumented) Latin American immigrants in the US (44). When sick, undocumented immigrants seek primarily cheap, low-quality healthcare resources before attending a healthcare center or a general hospital. They first tend to look for medical help in the immigrant shelters, and when this is not possible, they tend to self-medicate (45% of immigrants who had a health problem during past year), or go to drugstores where they get medicines without seeing a physician (11%), because they considered that health services are expensive and unfriendly for immigrants (Figure 1).

The absence of social support also weakens their health status, as there is no family member or friend to encourage them to go to a health center when necessary. Employment opportunities may rely on physical, mental, emotional, and spiritual wellbeing, and combined with the fact that migrants compete for obtaining the best jobs, the subsequent delay in health seeking is easily understandable. In many cases, migrants only look for medical assistance when they are completely incapacitated and suffer advanced disease (45–47). This behavior not only involves a delay in diagnosis, but also increases costs of treatment and a major public health burden (48).

Undocumented migrants are usually employed in the lowest paid jobs such as construction work, car washing, subway maintenance, food service, household cleaning, agriculture labor, and fruit picking. Especially in the construction sector, migrants are exposed to high labor risks, which are even increased because they are untrained for this kind of dangerous work and unprotected, as they have neither an accident nor a valid healthcare insurance in Mexico. Only 13.33% of the immigrants had a health insurance of any type in their home country.

Despite the fact that most migrants work in low-skilled and precarious, informal jobs, a daily wage in Nuevo Leon – the most competitive Mexican state with the highest per capita income after the capital city Mexico City and the highest average wage (49, 50) – may be equal to several days of pay in their home country. Although migrants identify economic factors as one of the main barriers to healthcare services, they may be unaware of the impact of education-related factors, such as knowledge and attitude, on their health-seeking behavior (51).

### Education, literacy, and health

**Figure 2:**
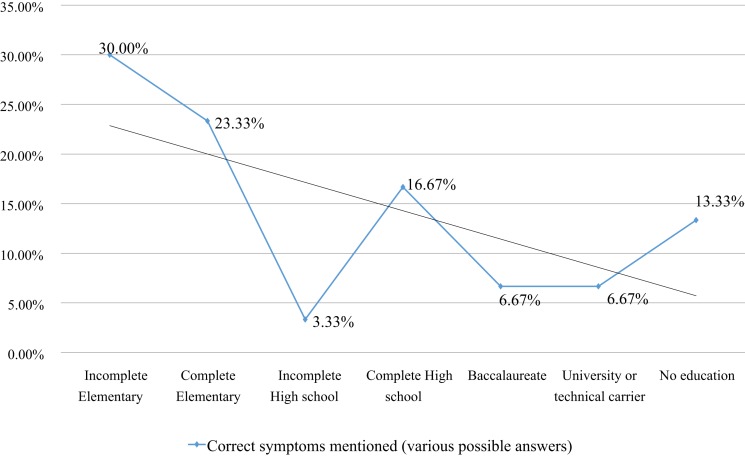
**Education level and TB symptoms knowledge**.

Previous studies on the impact of education on health practices in other Hispanic populations have shown that a high educational level is associated with better healthcare practices (52–54). In our sample, one-third had completed elementary school, 22.7% had completed high school level, and only 5.2% had studied at least one year of superior education (Table 1). Low literacy may be an obstacle in adherence to medical treatments, since the physician’s written or oral recommendations may result incomprehensive. For the same reason, information campaigns may result unsuccessful (55). Unexpectedly, in our study, immigrants with a lower level of education had better information on TB symptoms (*r* = 0.4; Figure 2), indicating that regular education may not be the main determinant of TB knowledge, which suggests that a specific personal experience may compensate for lack of school knowledge.

**Figure 3:**
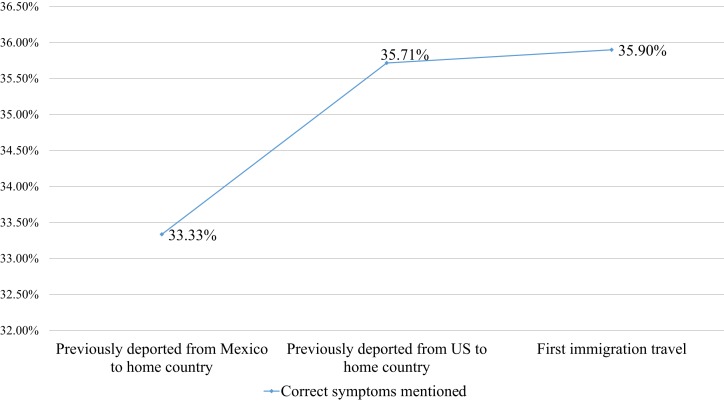
**Immigration experience and TB symptoms knowledge**.

Additionally, 25.33% of the sample had had a previous experience of being undocumented in the US, and 84% of those had undergone a deportation process. However, migrants with a previous deportation experience did not have more knowledge on TB (*r* = 0.8; Figure 3), even though a tuberculin test had been applied to them during the deportation process.

Considering that (1) deported migrants may intend another migration process, as evidenced by this study, (2) their knowledge on TB is insufficient (despite a previous deportation) process), and (3) their health status may be worse on a second or later intention of migration toward the US, we recommend health education, especially on TB, to be included in the deportation process. In Mexico, TB cases are concentrated at the borders and migration routes; and in the US, the US–Mexican border also demonstrates increased TB prevalence. In most countries, 10–15% of TB cases were related to undocumented migrants (24). Therefore, health issues of attending migrants may diminish contagious diseases and improve the health status of the general population (56).

### Inadequate information and wrong perceptions may lead to detection delays

Health access is also determined by the risk perception of contracting illnesses. Our results show an inadequate education and attitude toward health issues, especially TB. While 11 persons (14.7%) had been in contact with a TB patient and the infection was perceived as “serious” or “very serious” by most migrants (81.3%), there was confusion about TB symptoms and a shortage of knowledge of TB. This lack of concern is preoccupying, because it seems highly unlikely they were to seek healthcare in case of presenting TB symptoms. Once again, it underscores the need for culturally adequate, affordable, and accessible healthcare.

This low level of information is consistent with previous studies on different populations, such as Latinos migrants (57), immigrant farm workers (58), and Vietnamese refugees (59). The combination of a strong belief of not being infected, a 10–20 times higher exposure to TB, and little knowledge on TB symptoms forms an important reason for delayed TB detection in the migrant population delays a TB diagnosis (60–63).

For all the above reasons, adequate health education may (1) improve knowledge on TB among migrants, (2) stimulate appropriate health-seeking patterns, (3) lower risk factors, (4) increase compliance with treatment beyond the disappearance of symptoms, as is needed to treat TB, and (5) prevent the formation of resistant *Mycobacterium tuberculosis* strains (64). As personal health is not the main concern of many migrants, the use of incentives and enablers could contribute to stimulate the participation commitment to health-seeking behavior.

### Implications and recommendations

The increased relative risk of latent and active TB in the undocumented population of the Monterrey Metropolitan Area represents a health threat for the general population because of the frequent and prolonged interactions between the migrants and local inhabitants. Indeed, most migrants in our sample traveled through Mexico step-by-step, or city-by-city, and they frequently lived and worked for days, weeks, or longer in the cities they crossed. A shelter is considered a short-term lodging solution upon arrival to a new city, which is replaced by another accommodation for longer stays. The average stay of the migrants in our study in Mexico was 249.69 ± 1249.65 days (mean ± SD) (range: 1–11315 days). Furthermore, 71% of them would rather stay and live in Mexico than return to their home countries in case they could not cross the Mexican–US border. Almost three quarters considered the Monterrey Metropolitan Area to be a privileged place of residence because of job opportunities and a relatively good payment.

In summary, the combination of migrants’ vulnerability and inadequate health-seeking patterns, the length of their stay, and the lack of dedicated public policies may represent a health threat not only to the migrants, but also for public health in general. Our study confirms global evidence that transmission of infectious diseases, such as TB, may be intimately linked to migration issues (65–68). This constitutes a major difference with other vulnerable, but non-migrant populations, who may also suffer poverty and low educational attainment, but do have access to the social security system, for example, 77.1% of the Nuevo Leon inhabitants have social coverage, whereas undocumented migrants do not (69).

So far, there have been few publications on health issues among undocumented migrants. Further investigations on current health status and disease consequences should be encouraged. Our research provides a good starting point to identify broad outlines of public policy implications and recommendations. In addition to physical health issues, attention should be given to social-economical and emotional aspects, as the latter have a great impact on health-seeking behavior of migrants during the long, traumatic, and solitary experience of their often-violent journey.

Migrants’ health behaviors are the result of consciously planned decisions and thoughtless habits; the latter are probably harder to be modified than the former. A successful policy to improve migrants’ health status and diminish the threat to public health in general needs to go beyond the current bio-medical model toward a bio-psychosocial model. To improve efficiency and circumvent migrants’ self-exclusion from health services, migrants’ healthcare should be compatible with the immigrants’ specific characteristics: low level of health literacy, need of anonymity, and availability outside their working hours.

The migration process has a negative impact on health issues, which are reinforced by inequality between migrants and the general population in access to medical services. If securing public health would become an objective, the focus might be on the fundamental determinants of health through multi-sectoral policies involving both private and public sectors at local, national, and international levels. Preventive efforts are indispensable to foster health monitoring in the migrant population, and transnational teamwork is crucial to enforce cross-border cooperation to develop referral strategies and appropriate mechanisms for undocumented migrants beyond the local scale, and much before they arrive to the Mexican–US border.

Because of the harsh economic situation of migrants, healthcare has become a luxury for them because of its high financial cost, thus representing a powerful barrier to health-seeking behaviors. To ensure proper health access to migrants, a mechanism should be created to facilitate free access to health services, regardless of their legal status. The cost of these healthcare mechanisms should be fully covered by the local and binational authorities, given the potential implications for public health of not considering this highly vulnerable population (undetected diseases and infections, delay in diagnosis, increased costs of future treatments, transmission to healthy subjects) (70–72).

Future studies could implement Knowledge, Attitude, and Practices (KAP) analysis to deepen the understanding of the migrants’ values and beliefs on the one hand and a targeted screening on several border health issues on the other hand. Actually, our research team plans to start a TB-screening campaign in summer 2015, in order to estimate the burden of latent tuberculosis infection in undocumented immigrant and to eventually offer them prophylaxis before offering treatment (73), as many countries do (62, 74–77). Migrants might be an exposed-to-TB group, due to their social vulnerability (78, 79), but we cannot address this potential problem without measuring it first.

Likewise, we aim to measure the prevalence of DM in subsequent studies, as we detected that, although most individuals in our sample suffered poor nutrition, more than half (55%) were overweight or obese and only 44.7% had a normal BMI. This dichotomy is suggestive of a high prevalence of DM, even though only two migrants explicitly referred to suffer from DM, possibly due to an under-diagnosis of DM or a failure to detect it in this population.

In fact, the World Health Organization (WHO) announced a conditional recommendation of systematic testing of immigrants from high TB burden countries (Honduras had a TB prevalence of 74/100,000; Guatemala: 110/100,000; El Salvador: 48/100,000) (80), although, there is no clear evidence of benefits of systematically screening immigrant population (81). For this reason, we aim to generate more epidemiologic knowledge of TB burden among undocumented migrants and, afterward, on cost-effectiveness of TB-related interventions (education, detection, and treatment), in order to create better fitting strategies to migrants requirements and to prevent possible active disease (82–84).

We propose practical ways to improve health equality for migrants and diminish social exclusion through recommendations to policy-makers, like tracking and preventing disease strategies in immigrant communities, and educating and building communication; since the main reason for the spread of infections like TB is their unawareness (85). We expect that health education will increase adequate health-seeking patterns among (undocumented) migrants.

## Conflict of Interest Statement

The research was conducted in the absence of any commercial or financial relationships that could be construed as a potential conflict of interest.

## Abbreviations

AIDS, acquired immune deficiency syndrome; BCG, Bacillus Calmette–Guérin; DM, diabetes mellitus; HIV, human immunodeficiency virus infection; INM, National Institute of Migration; KAP, knowledge, attitude and practices; NGO, no governmental organization; SD, standard deviation; TB, tuberculosis; US, United States; WHO, World Health Organization.
